# Nanoparticles as Drug Delivery Systems in Intravesical Therapy for Non-muscle Invasive Bladder Cancer: A Narrative Review

**DOI:** 10.7759/cureus.91103

**Published:** 2025-08-27

**Authors:** Jan Górski, Witold Gajewski, Hubert Ziembicki, Weronika Pierudzka

**Affiliations:** 1 Hospital Medicine, University Clinical Hospital in Poznan, Poznań, POL

**Keywords:** drug delivery systems, intravesical therapy, nanomedicine, nanoparticles, nmibc

## Abstract

Non-muscle invasive bladder cancer (NMIBC) accounts for the majority of newly diagnosed bladder cancer (BC) cases and is known for its high recurrence rate after initial treatment. The current standard of care includes the transurethral resection of bladder tumor (TURBT), followed by adjuvant intravesical therapy. However, therapeutic outcomes of intravesical therapies are often limited by the physiological characteristics of the urinary bladder, including continuous urine production and voiding, as well as the presence of the bladder permeability barrier (BPB), which reduces drug retention time and limits therapeutic efficacy. In response to these limitations, nanoparticle-based (NP-based) drug delivery systems (DDS) have emerged as a promising tool to prolong intravesical drug retention, improve treatment efficacy, and minimize systemic toxicity.

This narrative review provides an overview of the available literature on NP-based DDS, with applications in the intravesical treatment of NMIBC, categorizing them into organic, inorganic, carbon-based, and composite-based nanoparticles (NPs). A comprehensive literature search was conducted using PubMed and Google Scholar (up to 2025), with Boolean combinations of terms related to NMIBC, NP-based DDS, intravesical therapy, and the various subtypes of NPs investigated in this review. Each NP subtype offers distinct advantages, such as improved mucoadhesion, controlled drug release, multifunctionality, and potential for targeted delivery. Preclinical studies have shown encouraging outcomes; however, there is a lack of clinical data due to concerns related to toxicity, cost, scalability, and regulatory approval. Continued research and well-designed clinical trials are crucial for validating the safety and efficacy of NP-based DDS. In the future, the successful integration of NP-based DDS into intravesical therapy of NMIBC could reduce recurrence rates and improve therapeutic outcomes.

## Introduction and background

Bladder cancer (BC) is a prevalent urological malignancy, with approximately 600,000 new cases diagnosed annually worldwide. It accounts for 3.1% of all newly diagnosed cancers and ranks as the ninth most common cancer in the world. Furthermore, it is responsible for 2.3% of all cancer-related deaths, placing it 13th in terms of cancer-associated mortality [1-3]. Most BC cases are classified as urothelial carcinomas, with 75% of patients presenting with non-muscle invasive bladder cancer (NMIBC) at the time of diagnosis [4]. The current standard of care for NMIBC involves the transurethral resection of bladder tumor (TURBT), followed by various forms of adjuvant intravesical therapies; however, this treatment approach is often inadequate due to high recurrence rates. According to Teoh et al. (2022), the five-year recurrence rates for NMIBC range from 31% to 78% [5].

One of the contributing factors to high recurrence rates is the limited visibility of small and/or flat lesions under white light TURBT, which can result in incomplete resection and residual disease [6]. Additionally, physiological aspects of urinary bladder function - such as continuous urine production and voiding - along with the bladder permeability barrier (BPB), decrease the effectiveness of intravesical therapies [7]. These factors contribute to high recurrence rates in NMIBC patients and have prompted the development of novel treatment strategies to enhance NMIBC management. Among these, nanoparticle-based (NP-based) drug delivery systems (DDSs) have emerged as a promising avenue.

**Figure 1:**
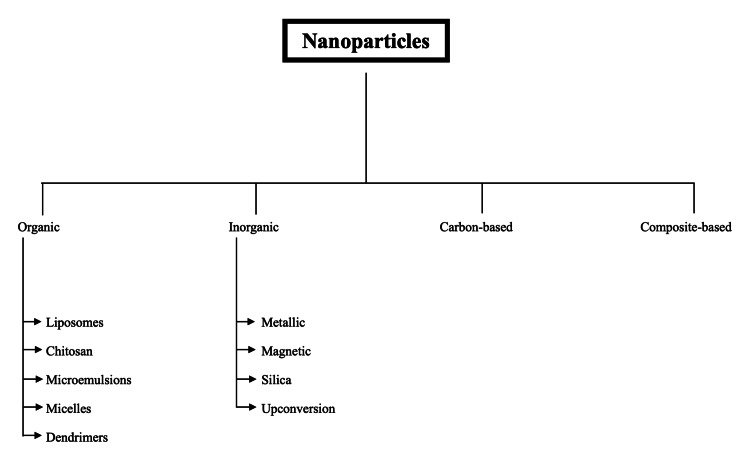
Nanoparticle types

Advances in nanotechnology have enabled the development of nanoparticles (NPs) capable of carrying therapeutic agents, including chemotherapeutics, immunotherapeutics, gene therapies, and photodynamic agents [8]. NPs are broadly categorized into four groups: organic NPs (liposomes, chitosan (CS), microemulsions, micelles, and dendrimers), inorganic NPs (metallic, magnetic, silica, and upconversion NPs), carbon-based NPs, and composite-based NPs (Figure 1). Each group possesses unique physicochemical properties, offering distinct therapeutic advantages and limitations [9]. This review aims to explore the application of NP-based DDSs in intravesical therapies for NMIBC.

## Review

Methods

Study Design

This narrative review aimed to provide an overview of the available literature on NP-based DDSs with applications in the intravesical treatment of NMIBC, categorizing NPs into organic, inorganic, carbon-based, and composite-based types. A comprehensive literature review was conducted using PubMed and Google Scholar, covering publications up to 2025, with particular emphasis on studies from 2015 to 2025. Older studies were included in cases where current data were limited or lacking.

The search strategy employed Boolean combinations of the following terms: (‘NMIBC’ OR ‘non-muscle invasive bladder cancer’ OR ‘bladder cancer’) AND/OR (intravesical therapy) AND/OR (‘nanoparticle-based drug delivery systems’ OR ‘nanoparticles’ OR ‘drug delivery systems’) AND/OR (‘liposomes’ OR ‘chitosan’ OR ‘microemulsions’ OR ‘micelles’ OR ‘dendrimers’ OR ‘metallic nanoparticles’ OR ‘magnetic nanoparticles’ OR ‘silica nanoparticles’ OR ‘upconversion nanoparticles’ OR ‘carbon-based nanoparticles’ OR ‘composite-based nanoparticles’).

The inclusion criteria encompassed peer-reviewed original research articles, reviews, scoping reviews, systematic reviews, meta-analyses, and preclinical and clinical studies focused on the design, application, and/or therapeutic evaluation of NP-based DDSs in intravesical therapies for NMIBC. Particular attention was given to identifying available clinical data and ongoing registered trials to capture the current stage of clinical translation. When clinical data were lacking or limited, secondary emphasis was placed on in vivo studies, and tertiary emphasis on in vitro studies. The exclusion criteria included studies focused on systemic nanotherapy and/or non-NP-based intravesical therapies. Additionally, reference lists of all eligible studies were manually reviewed to identify further relevant sources.

This review does not include a meta-analysis due to the substantial heterogeneity observed across the included studies, including differences in NP subtypes, experimental models, therapeutic agents, and outcome measures. This variability prevented meaningful statistical analysis and compromised the reliability of pooled estimates. Instead, the focus was placed on identifying recurring challenges, promising innovations, and gaps in current research to guide future investigations in NP-based DDSs for intravesical therapies of NMIBC.

Risk of Bias

As a narrative review, the lack of formal systematic review protocols and quality assessment frameworks introduces potential selection bias. Inclusion criteria were based on the judgment of the authors rather than standardized methodological criteria. Efforts were made to ensure comprehensive coverage of available literature through Boolean search strategies in PubMed and Google Scholar; however, sole reliance on these databases may have led to the exclusion of other relevant studies.

Furthermore, the considerable heterogeneity in NP subtypes, therapeutic agents, experimental models, and outcome measures across all included studies limited the ability to draw direct comparisons and conduct meta-analytic synthesis. As a result, this review focused on qualitative synthesis and thematic categorization rather than quantitative data evaluation (e.g., retention time, bladder wall penetration, or therapeutic responses).

The risk of publication bias cannot be excluded. Studies reporting positive and/or promising outcomes are more likely to be published and cited, thus skewing the evidence in favor of NP-based DDSs in intravesical therapies of NMIBC. Additionally, although the review prioritized literature from 2015 to 2025 to reflect current developments, older but still clinically relevant studies may have been underrepresented.

Lastly, interpretive bias may have been introduced through subjective summarization and emphasis on specific findings. The authors strived to present a balanced and comprehensive overview; however, the conclusions drawn should be interpreted with caution and viewed primarily as a foundation for further research rather than definitive clinical guidance.

Current treatment of NMIBC

TURBT is the primary therapeutic intervention for NMIBC [10]. Following TURBT, patient management is guided by post-operative risk stratification. Various risk stratification systems for NMIBC are in use, with the most widely recognized being those of the European Association of Urology (EAU), the American Urological Association (AUA), and the National Comprehensive Cancer Network (NCCN). According to the most precise of these, the EAU NMIBC 2021 scoring model, patients are categorized into low-, intermediate-, high-, and very high-risk groups based on tumor characteristics, incorporating both the World Health Organization (WHO) 2004/2016 and WHO 1973 grading systems [11].

The 2024 guidelines of the EAU recommend adjuvant intravesical therapy for most patients post-TURBT. Treatment options include intravesical chemotherapy, Bacillus Calmette-Guérin (BCG) immunotherapy, or a combination of both. Common chemotherapeutic agents used in intravesical therapies include gemcitabine, docetaxel, pirarubicin, epirubicin, and mitomycin C [12,13]. The standard treatment for carcinoma in situ (CIS) is intravesical BCG therapy, which reduces recurrence and progression. If BCG fails or the disease recurs, intravesical chemotherapy may be considered. Radical cystectomy (RC) is reserved for cases of BCG failure or high-risk disease when intravesical treatments are ineffective [14,15].

According to EAU guidelines, the treatment of patients with low-risk NMIBC typically involves a single, immediate instillation of intravesical chemotherapy. Intermediate-risk patients benefit from either one year of adjuvant chemotherapy or one year of full-dose BCG therapy, which includes an induction phase (six weekly instillations) followed by maintenance therapy (three weekly instillations at 3, 6, and 12 months). For high-risk patients, full-dose BCG therapy consists of an induction phase with six weekly instillations, followed by maintenance therapy with three weekly instillations at 3, 6, 12, 18, 24, 30, and 36 months.

In cases of failure to respond to BCG or disease recurrence, RC may be considered as an alternative therapeutic approach. In very high-risk cases, RC is the recommended treatment, with extended BCG therapy serving as an alternative if RC is contraindicated or declined [15,16].

Progression probabilities vary significantly among risk groups. According to Sylvester et al. (2021), the five-year progression probabilities under the WHO 2004/2016 system are 0.93%, 4.9%, 9.6%, and 40% for low-, intermediate-, high-, and very high-risk groups, respectively, and 0.57%, 3.6%, 11%, and 44% under the WHO 1973 system, respectively [11].

Despite established protocols, recurrence rates remain unacceptably high. Teoh et al. (2022) reported recurrence rates of 31% to 78% over five years, underscoring the need for innovative treatment strategies to reduce recurrence and progression [5]. In this context, NP-based DDSs present a promising solution by potentially reducing recurrence rates and improving therapeutic outcomes.

Urinary bladder anatomy and physiology

The urinary bladder is a distensible, hollow organ responsible for the temporary storage and subsequent excretion of urine. Urine produced by the kidneys enters the bladder via the ureters and accumulates until it reaches a volume of approximately 150-200 mL, at which point the sensation of urinary urgency arises. A stronger signal is generated at 400-600 mL, initiating micturition through the urethra. This process is regulated by coordinated interactions between the bladder musculature and the nervous system [17].

**Figure 2:**
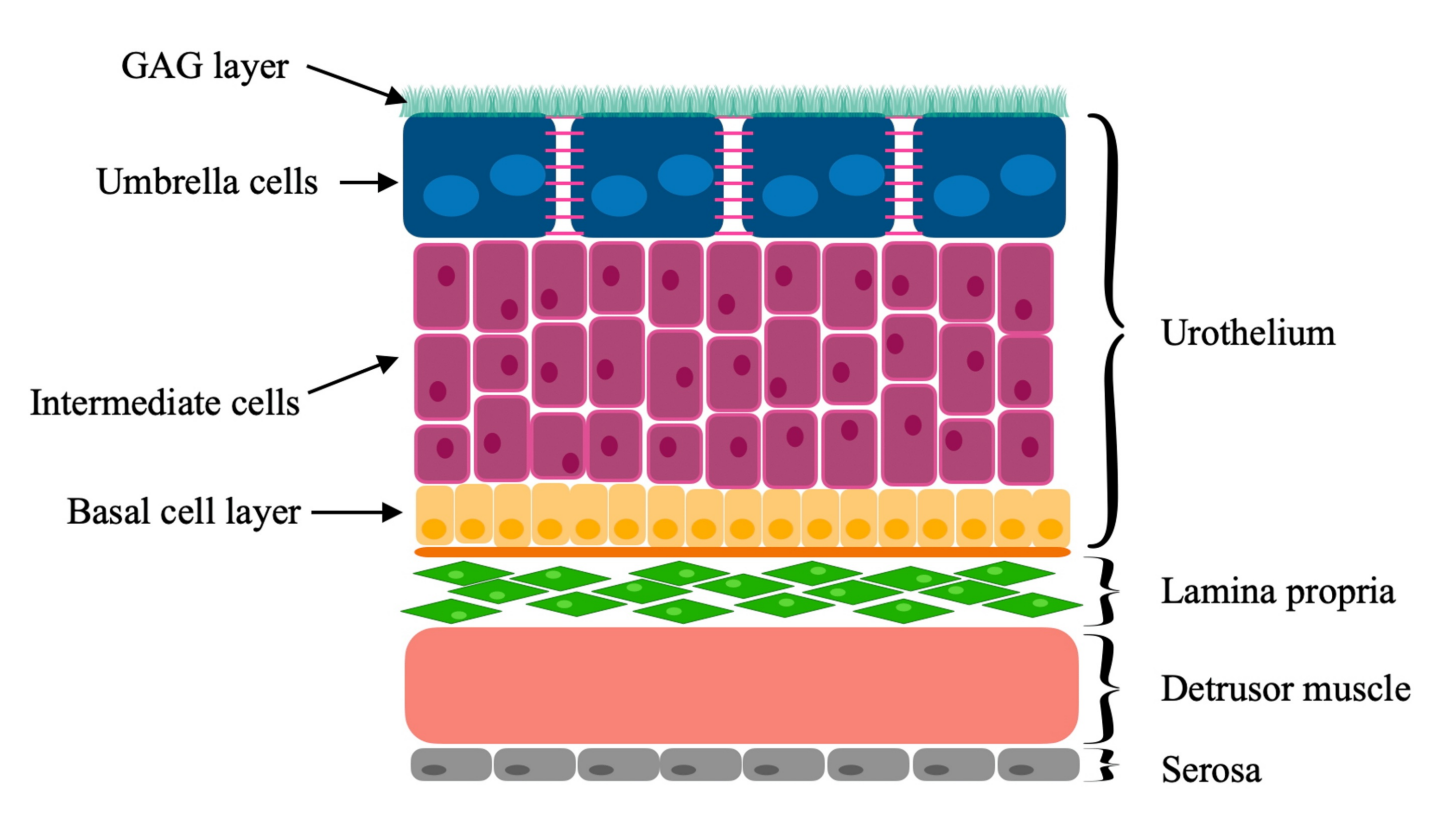
Bladder wall scheme GAG, glycosaminoglycan

The urinary bladder’s excretory function necessitates impermeability to various substances present in urine. Histologically, the walls of the urinary bladder are composed of the urothelium (transitional epithelium), lamina propria, detrusor muscle, and serosa (Figure 2). The urothelium serves as a critical component of the BPB, which functions similarly to the blood-brain barrier, but with even greater impermeability - thereby preventing the reabsorption of potentially toxic urinary components into systemic circulation [18].

The barrier function of the urothelium is maintained by specialized structural features. Intercellular junctions between superficial umbrella cells, along with desmosomes and cytoskeletal filament connections to the basal membrane, form a highly cohesive and impermeable cellular layer [19]. On the apical surface, umbrella cells are covered with scallop shell-shaped plaques, which form an asymmetric membrane, further reinforcing the barrier [20]. Additionally, a mucinous layer composed of negatively charged glycosaminoglycans (GAGs) covers the urothelial surface. This layer not only prevents the adhesion of harmful substances but also impedes their penetration into the bladder wall [21].

These structural and physiological adaptations, while essential for bladder protection, create formidable challenges for intravesical therapy. Continuous urine production leads to rapid dilution and washout of administered drugs, significantly decreasing drug retention time and limiting their therapeutic effect [7,22]. Moreover, the BPB limits the absorption of therapeutic agents across the urothelium, necessitating repeated drug instillation and increasing the risk of systemic toxicity. Consequently, these factors decrease the efficacy of conventional intravesical therapy.

To address these limitations, research efforts have increasingly focused on advanced drug delivery strategies. Among them, NP-based DDSs have gained attention for their ability to prolong drug retention, improve treatment efficacy, and minimize systemic toxicity.

Organic-based NPs

Organic-based NPs are constructed from organic materials and exclude carbon-based NPs. They rely on noncovalent intermolecular interactions to form diverse structures, such as liposomes, CS, microemulsions, micelles, and dendrimers. Each subgroup possesses distinct physicochemical characteristics that make them suitable for specific clinical applications [9].

Liposomes

Liposomes are among the most studied organic NPs. They are artificially synthesized nanovesicles composed of phospholipid bilayers capable of encapsulating both hydrophilic and hydrophobic substances, making them ideal carriers for a variety of drugs, proteins, and nucleotides [23,24]. Their clinical utility is supported by advantageous properties such as high bioavailability, biodegradability, biocompatibility, high encapsulation rates, a stable structure, and a relatively simple manufacturing process [8,25,26]. Drug release from liposomes can be triggered by environmental stimuli, leading to the classification of liposomes into six subgroups: thermosensitive, pH-sensitive, ultrasound-sensitive, enzyme-triggered, magnetic field-sensitive, and ligand-targeted liposomes [27,28].

A notable application of liposomes as NP-based DDSs in intravesical therapies of NMIBC includes the encapsulation of the BCG cell wall skeleton (BCG-CWS), which is the primary immunogenic component of the standard BCG immunotherapy. Due to its insolubility, BCG-CWS tends to aggregate, reducing its uniform distribution in the bladder and decreasing therapeutic efficacy. Whang et al. (2020) demonstrated that liposomal encapsulation of BCG-CWS improves solubility, prevents aggregation, and enhances stability in an orthotopic BC mouse model, suggesting significant therapeutic potential [29].

In a different study, Oefelein et al. (2020) reported the successful use of a proliposomal formulation of paclitaxel (PTX) in intravesical therapy of NMIBC. This formulation was shown to achieve deep detrusor muscle penetration, intravesical persistence of up to 72 hours, and high urinary concentrations, with minimal systemic exposure. No recurrence was observed during 12 months of follow-up in low- to intermediate-risk NMIBC patients [30]. However, despite promising outcomes, liposomal DDSs still face challenges, such as high production costs and off-target accumulation, limiting broader clinical application [8]. Continued advancements in nanotechnology are essential to overcome these barriers and facilitate the widespread adoption of liposomal DDSs in NMIBC therapy.

Chitosan (CS)

CS is another promising organic NP derived from the partial deacetylation of chitin, a naturally occurring compound found in fungal cell walls, certain bacteria, seaweed, and arthropod exoskeletons. Its biodegradability, low toxicity, and biocompatibility have made it attractive for biomedical applications, particularly drug delivery [31]. Structurally, CS consists of β-(1,4)-D-glucosamine and N-acetyl-D-glucosamine units, with reactive hydroxyl and amino groups that enable cross-linking and facilitate mucoadhesion. Its positive charge allows strong interactions with negatively charged mucosal surfaces, enhancing drug retention and penetration [21].

Liu et al. (2018) demonstrated the efficacy of PTX-loaded CS nanosuspensions (PTX/CS NSs) in intravesical therapy of NMIBC in a rat model study. PTX/CS NSs adhered to the mucin layer, enabling controlled PTX release over 10 days and effectively inhibiting tumor proliferation with low toxicity in both in vitro and in vivo models [32]. CS has also been employed as a carrier for indole-3-carbinol (I3C), a natural compound with antiproliferative and pro-apoptotic properties. When combined with CS NPs, I3C significantly reduced BC cell viability after 24 hours compared to the control group, indicating enhanced therapeutic potential compared to free I3C [33,34]. Another study by Xu et al. (2020) utilized CS to deliver gambogic acid, a potent anticancer agent with poor solubility and high systemic toxicity. Their CS-based prodrug system allowed prolonged retention and reactive oxygen species (ROS)-triggered release, showing strong anticancer effects with minimal urothelial toxicity in animal models [35,36]. These findings underscore the potential of CS NPs to enhance drug retention, mucoadhesion, and targeted delivery in NMIBC therapy.

Microemulsions

Microemulsions represent another class of organic NP-based DDSs, composed of two immiscible liquid phases stabilized by surfactants, forming nanodroplets ranging from 10 to 200 nm in size [37,38]. Their low density, small droplet size, and inclusion of surfactants enhance urothelial permeability, making them suitable for delivering both hydrophobic and hydrophilic drugs. Chen et al. (2020) explored a viscous microemulsion containing cisplatin and gemcitabine to improve bladder wall adherence and drug retention. Compared to aqueous gemcitabine, the microemulsion achieved longer drug retention and higher drug concentrations in the bladder, without causing urothelial toxicity in a rat model study [39,40]. These features suggest microemulsions could significantly improve the efficacy of intravesical chemotherapy by enhancing drug availability and minimizing systemic exposure.

Micelles

Micelles are an assembly of surfactant molecules organized into a hydrophobic core and a hydrophilic shell. The hydrophobic core facilitates the encapsulation of poorly soluble drugs, while the hydrophilic shell improves solubility and stability [41,42]. Degradable hyperbranched polyglycerols (dHPGs), known for their biodegradability and low cytotoxicity, have been used to form micelles for intravesical delivery. Mugabe et al. (2009) encapsulated PTX in dHPG-based micelles and compared them to Taxol (PTX in Cremophor-EL/ethanol) in an animal model experiment. Although Taxol exhibited slightly higher in vitro cytotoxicity toward BC cells, the dHPG-based micelles demonstrated superior tumor inhibition in vivo, with better tolerability - supporting their application in NMIBC treatment [43,44].

Dendrimers

Dendrimers are highly branched synthetic macromolecules with a central core and multiple functionalized surface groups, allowing for precise structural control and chemical modification. These properties enable high drug-loading capacity, increased solubility, and selective targeting [45]. Polyamidoamine (PAMAM) dendrimers are among the most widely studied [46,47]. Qiu et al. (2017) evaluated a polyethylene glycol-PAMAM-doxorubicin (PEG-PAMAM-DOX) conjugate in an NMIBC animal model study. Compared to free DOX, PEG-PAMAM-DOX achieved approximately fivefold greater tumor reduction and exhibited minimal urothelial toxicity, highlighting the advantages of dendrimer-based drug delivery [48].

Inorganic-based NPs

Inorganic-based NPs are composed of non-carbon materials and exhibit diverse morphologies, including spherical, rod-shaped, or amorphous forms. These NPs can be categorized into four primary groups: metallic, magnetic, silica-based, and upconversion NPs. Each category possesses distinct physicochemical characteristics, enabling specific applications in cancer therapy, including the treatment of NMIBC [9].

Metallic NPs

Metallic NPs typically range from 1 to 100 nm in size and are significantly smaller than many other NPs, allowing for enhanced cellular uptake and deeper tumor penetration. Their high surface-area-to-volume ratio facilitates efficient drug loading and delivery [49,50]. Cancer cell targeting is achieved by two main mechanisms. The first is passive targeting, through the enhanced permeability and retention effect, in which the leaky vasculature and impaired lymphatic drainage of tumors facilitate NP accumulation [49,51]. The second is active targeting, in which ligands, such as peptides or antibodies, are bound to the NP surface, enabling specific interactions with tumor-associated receptors. This strategy allows for precise delivery, minimizing off-target toxicity and improving therapeutic efficacy.

Gold NPs (AuNPs) have gained considerable interest due to their low toxicity, surface tunability, and unique optical properties, such as surface plasmon resonance (SPR). The negatively charged surface of AuNPs allows for easy functionalization with ligands or therapeutic agents. Additionally, their accumulation in tumor tissue induces observable colorimetric changes via SPR, enabling their use in visual diagnostics [52,53]. Yafout et al. (2021) reported that AuNPs effectively enhanced drug delivery in cancer therapy, reducing the required therapeutic dose and associated toxicity [54]. However, there is still a lack of clinical studies focusing on the application of AuNPs as DDSs in NMIBC treatment.

Silver NPs (AgNPs) exhibit cytotoxic effects toward cancer cells through multiple mechanisms, including oxidative stress, DNA damage, and cell membrane disruption. These effects ultimately induce apoptosis or necrosis in tumor cells. Ferreira et al. (2020) demonstrated that treatment with AgNPs significantly reduced tumor size in NMIBC in vivo animal models [55]. Despite these encouraging findings, clinical applications remain limited due to insufficient data on human toxicity and long-term safety. More robust clinical research is required before AgNPs can be integrated into NMIBC therapy [56].

Magnetic NPs

Unlike other NPs, magnetic NPs are not defined by their structural properties but rather by their ferromagnetic traits. The ability to be controlled by external magnetic fields makes them ideal tools for targeted drug delivery. Therapeutic agents can be adsorbed onto or encapsulated within magnetic NPs, which can then be directed to tumor sites with an external magnet, enhancing local drug concentration and minimizing systemic exposure [57]. Zakaria et al. (2015) demonstrated that magnetically guided iron oxide NPs (FeNPs) improved drug efficacy when used on T24 BC cells by increasing local drug concentrations compared to controls [58,59]. Additionally, FeNPs have shown promise in diagnostics, facilitating tumor visualization via magnetic resonance imaging (MRI) and enabling magnetic field-induced hyperthermia, which improves therapeutic outcomes by damaging cancer cells [60]. While preclinical data is promising, clinical validation is still needed to confirm their applicability in human NMIBC treatment.

Silica NPs

Silica NPs, often referred to as mesoporous silica NPs, are composed of pure silica and possess a porous structure that allows for the encapsulation and controlled release of therapeutic agents. Their size typically ranges from 10 to 300 nm, with variable pore size and density tailored to the characteristics of the encapsulated drugs. Drug release is typically triggered by internal or external stimuli such as changes in pH or enzymatic activity, ensuring precise and localized delivery to tumor sites [61]. Although silica-based DDSs have been extensively studied in other cancer types, no data were found specific to NMIBC treatment.

Upconversion NPs

Upconversion NPs represent a unique class of NPs composed of lanthanide-doped materials, typically containing ytterbium (Yb³⁺) and thulium (Tm³⁺) ions, which absorb near-infrared (NIR) light and emit higher-energy visible (VL) or ultraviolet (UV) light. These NPs are not used independently, but are typically functionalized with photosensitizers or other therapeutic agents [62]. Wang et al. (2011) developed PEGylated upconversion NPs conjugated with DOX, where drug release was triggered by acidic pH - a characteristic feature of tumor microenvironments. In this case, upconversion NPs enabled real-time monitoring of NP localization and therapeutic distribution [63]. However, in the context of NMIBC, upconversion NPs have primarily been studied for diagnostic purposes rather than direct therapeutic use. Further clinical research is essential to unlock their full potential in intravesical therapies of NMIBC.

Carbon-based NPs

Carbon-based NPs are synthetic nanostructures composed primarily of carbon, designed to transport therapeutic agents, such as chemotherapeutics, directly to targeted cells. These systems utilize ligand-mediated targeting to enhance specificity and reduce off-target toxicity. Carbon-based NPs include a wide variety of structures, such as quantum carbon dots (C-dots), carbon nanotubes (CNTs), carbon nanosheets (CNs), and carbon nanoribbons (CNRs), typically ranging in size from 1 to 100 nm. Their versatility stems from the ability to modify key properties through surface functionalization, including optical characteristics, drug loading efficiency, biocompatibility, immunogenicity, and targeting capabilities [64,65]. These modifications improve delivery efficiency by improving drug retention. Moreover, carbon-based NPs possess intrinsic antitumor activity through the generation of ROS, ultimately leading to tumor cell death. However, this mechanism also contributes to the cytotoxicity associated with carbon-based NPs, posing a challenge in their clinical use.

A promising application of carbon-based NPs in NMIBC therapy was demonstrated by Menilli et al. (2021), who investigated the phototoxic potential of graphene oxide and graphene quantum dots for the delivery of tetracationic porphyrins in T24 BC cells. The primary mechanism of action was ROS-mediated photocytotoxicity. Their study found that the carbon-based NPs improved porphyrin stability in aqueous environments and prolonged drug retention. Moreover, they suggested that surface functionalization could be used to specifically target receptors in BC, such as epidermal growth factor receptor (EGFR), human epidermal growth factor receptor 2 (HER-2), and fibroblast growth factor receptor 3 (FGFR-3), thereby increasing the efficacy and safety of the therapy [66].

Another example involves the use of single-walled carbon nanotubes (SWNTs) as carriers for pirarubicin in intravesical therapy of NMIBC. Chen et al. (2012) conducted a rat model study to evaluate the efficacy of pirarubicin-loaded SWNTs. Compared to pirarubicin alone, the pirarubicin-loaded SWNT formulation demonstrated higher tumor inhibition rates, both in vitro and in vivo. Importantly, while the pirarubicin group exhibited adverse effects such as hematuria, vomiting, and increased urinary frequency, the pirarubicin-loaded SWNT group experienced no observable side effects, indicating improved tolerability and safety [67]. These findings support the potential of SWNT-based DDSs to enhance therapeutic efficacy while reducing toxicity in NMIBC treatment.

Despite these promising applications, a major limitation of carbon-based NPs is their variable and often unpredictable toxicity. Their adaptability allows for a wide range of physical and chemical modifications, but this also results in a broad toxicity spectrum - from minimal to severe. Complications may include DNA damage, oxidative stress, apoptosis, pulmonary toxicity (fibrosis, pneumonitis, and granulomas), cardiopulmonary complications, and reproductive toxicity. Francis and Devasena (2018) highlighted these concerns and emphasized the lack of comprehensive data due to the relatively recent integration of nanotechnology into biomedical research. They stressed the need for rigorous safety assessments before carbon-based NPs can be considered viable for clinical use in NMIBC therapy [68].

Overall, carbon-based NPs offer exceptional potential in enhancing drug delivery specificity and prolonging drug retention. However, their clinical translation is currently limited by unresolved toxicity concerns and insufficient long-term safety data. Future studies must focus on optimizing biocompatibility, refining targeting strategies, and conducting comprehensive in vivo toxicity assessments to ensure safe and effective clinical application.

Composite-based NPs

Composite-based NPs are materials made from a combination of two or more types of NPs, or the combination of NPs with other larger, structurally complex materials, such as metals, ceramics, or polymers. By merging the advantageous features of individual components, composite-based NPs overcome the limitations inherent to single-component NPs. This approach allows for the customization of particle morphology and functionality, thereby enhancing performance and introducing new capabilities [9,69].

An example of composite-based NP application in NMIBC therapy is provided by Sun et al. (2022), who developed a multifunctional DDS composed of FeNPs, β-glycerophosphate, and CS, used for the intravesical administration of pirarubicin in a rat model study. This composite NP system exhibited multiple favorable features, including magnetic responsiveness, mucoadhesion, and sustained drug release. The system achieved prolonged bladder wall attachment and magnetic targeting, with retention exceeding 72 hours. Additionally, it demonstrated strong degradation characteristics and superior antitumor effects in both in vitro and in vivo models, compared to pirarubicin monotherapy [70].

Another notable rat model study by Suo et al. (2019) investigated the use of magnetic multiwalled carbon nanotubes (mMWCNTs) for the controlled intravesical release of epirubicin in NMIBC treatment. This composite system aimed to improve drug retention post-TURBT and enhance therapeutic outcomes. The mMWCNT-based formulation facilitated extended bladder retention through magnetic guidance and showed significantly greater cytotoxicity and antiproliferative effects in BC cells, both in vitro and in vivo, compared to conventional epirubicin treatment [71].

Overall, composite-based NPs represent a versatile and promising platform for NMIBC therapy. By harnessing the synergistic properties of multiple materials, these systems enable more precise targeting, prolonged intravesical drug retention, and controlled drug release. Although preclinical studies have yielded encouraging results, the transition to clinical application will require extensive trials to evaluate safety and efficacy. Continued innovation in composite NP design holds significant potential to improve outcomes in NMIBC management. A summary of NP characteristics and study outcomes is provided in Table 1.

**Table 1:** Summary of NP characteristics and study outcomes NP(s), Nanoparticle(s); NMIBC, Non-muscle Invasive Bladder Cancer; BCG-CW, Bacillus Calmette-Guérin Cell Wall; PTX, Paclitaxel; CS, Chitosan; NS(s), Nanosphere(s); I3C, Indole-3-Carbinol; ROS, Reactive Oxygen Species; dHPG, Degradable Hyperbranched Polyglycerol; PEG, Polyethylene Glycol; PAMAM, Polyamidoamine; DOX, Doxorubicin; Au-NPs, Gold Nanoparticles; Ag-NPs, Silver Nanoparticles; FeNPs, Iron Oxide Nanoparticles; SWNTs, Single-Walled Carbon Nanotubes; mMWCNTs, Magnetic Multiwalled Carbon Nanotubes

NP Types	NP Subtypes	Characteristics	Study Outcomes	Authors
Organic NPs	Liposomes	Encapsulate both hydrophilic and hydrophobic drugs; high bioavailability, biodegradability, biocompatibility, encapsulation rates, and stable structure; possibility for stimuli-mediated triggered release.	Increased solubility and stability, as well as decreased aggregation of BCG-CW; deep detrusor muscle penetration, intravesical retention time of up to 72h, and high urinary concentrations of paclitaxel proliposomal formulation.	Whang et al. (2020) [29]; Oefelein et al. (2020) [30]
	Chitosan	High biodegradability, biocompatibility, and low toxicity; cross-linking and mucoadhesive properties.	Controlled release over 10 days and inhibited tumor proliferation with low toxicity of PTX/CS NSs; inhibited tumor cell viability of I3C-CS; prolonged retention and ROS-triggered release of gambogic acid-CS.	Liu et al. (2018) [32]; Melo et al. (2021) [34]; Xu et al. (2020) [36]
	Microemulsions	Delivery of both hydrophobic and hydrophilic drugs; enhanced urothelial permeability.	Improved bladder wall adherence and drug retention, as well as higher drug concentrations of cisplatin-gemcitabine microemulsion.	Chen et al. (2020) [40]
	Micelles	Encapsulation of poorly soluble drugs; improved solubility and stability.	Improved in vivo tumor inhibition of paclitaxel dHPG-based micelles.	Mugabe et al. (2009)
	Dendrimers	High drug-loading capacity, increased solubility, and selective targeting.	Improved tumor inhibition of PEG-PAMAM-DOX.	Qiu et al. (2017) [48]
Inorganic NPs	Metallic	Efficient drug loading and delivery; passive and active modalities for targeting cancer cells; applications in visual diagnostics.	Lack of clinical studies focusing on Au-NPs in NMIBC treatment; significant reduction in tumor size of Ag-NPs.	Ferreira et al. (2020) [55]
	Magnetic	Ferromagnetic traits; targeted delivery via external magnetic field manipulation; applications in visual diagnostics.	Increased local drug concentrations of FeNPs.	Zakaria et al. (2015) [59]
	Silica	Stimuli triggered controlled release; variable pore size and density tailored to the characteristics of the encapsulated drugs.	Lack of clinical studies focusing on silica-based NPs in NMIBC treatment.	-
					Upconversion	Stimuli triggered controlled release; applications in visual diagnostics.	pH-triggered drug release and real-time monitoring of PEGylated upconversion NPs conjugated with DOX.	Wang et al. (2011) [63]
	Carbon-based NPs	-	Possibility for modification of key properties through surface functionalization, including optical characteristics, drug loading efficiency, biocompatibility, immunogenicity, and targeting capabilities; anti-tumor activity through ROS generation.	ROS-mediated photocytotoxicity through delivery of tetracationic porphyrins by graphene oxide and graphene quantum dots; higher tumor inhibition rates both in vitro and in vivo of pirarubicin-loaded SWNTs.	Menilli et al. (2021) [66]; Chen et al. (2012) [67]
Composite-based NPs	-	Customization of particle morphology and functionality, enhancing performance and introducing new capabilities.	Prolonged bladder wall attachment, magnetic targeting, retention exceeding 72 hours, strong degradation characteristics and enhanced antitumor effects of composite-based NPs based on FeNPs, β-glycerophosphate, and CS; extended bladder retention through magnetic guidance, and significantly greater cytotoxicity and antiproliferative effects in vitro and in vivo of epirubicin loaded mMWCNTs.	Sun et al. (2022) [70]; Suo et al. (2019) [71]

Key findings

NP-based DDSs can overcome bladder-specific challenges, such as drug washout and the BPB. Different classes of NPs - including organic, inorganic, carbon-based, and composite-based - offer unique advantages, including mucoadhesion, controlled release, imaging capabilities, and surface functionalization, allowing for multifunctionality. While preclinical studies show encouraging results, clinical translation is still constrained by toxicity, scalability, and regulatory approval.

## Conclusions

NMIBC represents a complex therapeutic challenge due to its high rates of recurrence and progression. Current intravesical treatment in NMIBC therapy faces limitations such as drug washout, poor urothelial penetration, and inadequate targeting possibilities. NP-based DDSs have emerged as a promising strategy to address these shortcomings by enhancing drug stability, retention, penetration, and specificity. Organic NPs, such as liposomes and CS, have strong mucoadhesive properties and increase intravesical drug retention. Inorganic NPs, such as metallic and magnetic NPs, offer precise targeting and multifunctional capabilities, including imaging and external control. Carbon-based NPs demonstrate high drug-loading capacity and intrinsic anticancer activity, while composite-based NPs combine traits from multiple single-component NPs, for improved performance.

Despite substantial preclinical progress, the translation of NP-based DDSs into routine clinical practice is still limited by concerns regarding toxicity, scalability, and regulatory approval. Future research must prioritize in vivo validation and optimize NP formulations for human use, with particular emphasis on long-term toxicity evaluation. With continued interdisciplinary innovation and clinical investigation, NP-based DDSs have the potential to revolutionize intravesical therapy, reducing recurrence rates and improving therapeutic outcomes in patients with NMIBC.
